# The Mukbang (Eating Broadcast) Paradox: Divergent Associations of Viewing Frequency with Improved Dietary Balance and Impaired Moderation in Korean Adults

**DOI:** 10.3390/nu18091478

**Published:** 2026-05-06

**Authors:** Ahyoung Yun, Hyein Jung, Byungmi Kim, Yoonjoo Choi

**Affiliations:** 1 Division of Cancer Prevention, National Cancer Control Institute, National Cancer Center, Goyang-si 10408, Gyeonggi-do, Republic of Korea; yay1210@ncc.re.kr (A.Y.); hi.jung@ncc.re.kr (H.J.); 2 Department of Public Health & AI, Graduate School of Cancer Science and Policy, National Cancer Center, Goyang-si 10408, Gyeonggi-do, Republic of Korea

**Keywords:** mukbang, food media, dietary quality, dietary behavior, impulsive eating

## Abstract

**Background:** Mukbang, a popular digital content genre where hosts consume large quantities and diverse foods on screen, has gained widespread popularity worldwide. Despite its influence, the association of mukbang viewing with dietary quality and behaviors in Korean adults remains unclear. This study aimed to investigate the association between mukbang viewing frequency and dietary quality and behaviors in Korean adults. **Methods:** The cross-sectional study examined data from a National Cancer Center survey involving 1210 Korean adults aged 20–64 years. Participants were categorized by mukbang viewing frequency and assessed using the revision of Nutrition Quotient for Korean Adults (NQ-2021), which includes three domains: Balance, Moderation, and Practice. Additionally, self-reported changes in eating behaviors, such as dining out, delivery, or instant food consumption, and impulsive eating, were investigated. Multivariable logistic regression was used to analyze these associations, adjusting for sociodemographic variables. **Results:** Compared with non-viewing, more frequent mukbang viewing was significantly associated with higher odds of being in a better grade in the Balance domain (≥5 times/week: OR = 2.88; 95% CI: 1.57–5.30), albeit with lower odds of being in the Moderation domain (≥5 times/week: OR = 0.21; 95% CI: 0.12–0.38). No significant differences were found in the Practice domain total score. Additionally, more frequent viewers also exhibited higher odds of increased dining out, delivery, or instant food consumption (≥5 times/week: OR = 3.24; 95% CI: 1.72–6.08), and impulsive/binge eating (≥5 times/week: OR = 2.80; 95% CI: 1.55–5.06). Interestingly, the “3–4 times/week viewing” group generated the highest odds of decreased dining out, delivery, or instant food consumption (3–4 times/week: OR = 3.55; 95% CI: 1.42–8.90). **Conclusions:** Mukbang viewing frequency is associated with both beneficial and detrimental dietary behaviors among adults. This study’s findings highlight mukbangs’ influence and the need for further research and public health strategies to maximize its potential benefits.

## 1. Introduction

Mukbang, a portmanteau derived from the Korean words for “eating” (meokneun) and “broadcast” (bangsong), is a type of digital content in which hosts eat a large amount or a wide variety of food, commonly shared via live or recorded streaming platforms [1]. Initially popularized in South Korea, mukbang has become a global phenomenon, attracting a growing audience across cultures and age groups through digital platforms such as YouTube [2]. Despite its global expansion, mukbang remains one of the most frequently viewed video genres among Korean viewers, particularly via online video platforms [3,4].

Several factors explain the rising popularity of mukbang. The visual presentation of food coupled with the host’s eating behavior, sounds, and expressions provides viewers with a sense of enjoyment and satisfaction [4,5,6]. This multisensory experience may also evoke feelings of satiety and vicarious fulfillment, as if the viewers themselves were eating [7,8]. For many, mukbang also serves as a “meal mate” substitute, helping reduce feelings of loneliness, especially in single-person households [1,7]. By watching hosts share their daily lives and interacting through comments, viewers often develop a sense of emotional connection and copresence [6,7,9]. In addition, numerous mukbang videos include cooking demonstrations or introduce new foods and restaurants, offering viewers useful tips and information while also enhancing the entertainment value of the content [4,10].

Considering that mukbang content focuses on food and eating, it has the potential to shape viewers’ eating behaviors and food-related decisions [11]. Mukbang content typically features the consumption of energy-dense, nutrient-poor foods and often involves excessive eating, potentially contributing to adverse health outcomes [1]. Several studies have reported that mukbang viewing among adolescents is associated with increased consumption of unhealthy foods, such as fast food, sugary drinks, coffee, and late-night snacks [12,13]. It has also been linked to poor dietary patterns (e.g., frequently eating out), lower Nutrition Quotient (NQ) scores [14], and obesity [4]. A higher frequency of mukbang viewing has been associated with increased dining out, delivery, or instant food consumption among Korean college students [15]. Additionally, in adults, a longer viewing time has also been associated with increased consumption of delivery food and late-night meals [11]. While certain studies have examined mukbang’s dietary effects in adults, most existing research has concentrated on adolescents. Consequently, evidence regarding its influence on eating behaviors in the general adult population remains limited.

Adults differ from adolescents in that their dietary habits are more established, and their eating behaviors may have greater implications for long-term health outcomes. Recent surveys in Korea indicate that mukbang constitutes a significant proportion of the online content consumed by middle-aged adults, with 37–46.8% of YouTube users aged 19–69 years reporting that they watch mukbang content [16], underscoring the need to examine its impact beyond adolescent and college-aged populations. Understanding this association may help characterize mukbang-related eating behaviors in adults and provide evidence to inform socio-nutritional intervention strategies, including nutrition education and guidance on digital food content use.

Therefore, this study aimed to investigate the association between mukbang viewing frequency and dietary behaviors in Korean adults aged 20–64 years. Specifically, we examined this relationship using the NQ-2021 and self-reported behavioral outcomes (e.g., increased dining out or impulsive eating) by comparing viewers and non-viewers. This study hypothesized that mukbang viewing frequency would be associated with dietary quality and behaviors.

## 2. Materials and Methods

### 2.1. Study Design

This cross-sectional study used data from an online survey conducted by the Division of Cancer Prevention, National Cancer Control Institute, National Cancer Center. The survey was conducted from 10 July 2024 to 23 July 2024, to investigate the relationship between mukbang viewing and dietary behaviors. The survey questionnaire included the following items: basic sociodemographic factors, mukbang viewing, the NQ score, and eating behaviors. This study was approved by the Institutional Review Board (IRB) of the National Cancer Center Korea (IRB Number: NCC2024-0161), and all study procedures were conducted in accordance with the Declaration of Helsinki. Informed consent was obtained from all participants prior to their participation in the survey.

### 2.2. Study Population

The study population consisted of Korean adults aged 20 to 64 years, recruited via an online panel provided by a professional survey company. Invitations were distributed through email or mobile notifications containing a survey link. To ensure representativeness, stratified quota sampling was employed based on age, sex, and region (17 administrative divisions) using population distribution data from the 2024 Korean national statistics.

A total of 1270 participants were recruited based on pre-specified quotas for food-related content-viewing status (1:1 ratio of viewers to non-viewers), age, sex, and region. Data collection continued until all target quotas were strictly fulfilled. During the recruitment process, individuals were excluded if they did not meet the age criteria (n = 418) or provided incomplete responses (n = 461). Additionally, access to the survey was automatically closed for individuals who attempted to participate after their specific demographic quota had already been reached.

**Figure A1:**
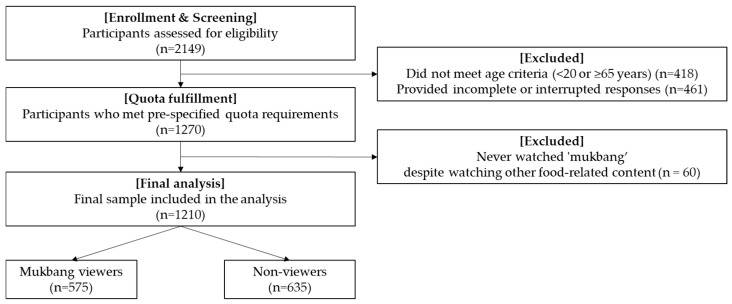
Flow chart of study population.

Among the 1270 participants who met the recruitment quotas, 60 individuals in the viewer group were excluded because they exclusively watched other food-related content (e.g., cooking or drinking shows) but reported never watching mukbang. Consequently, a final sample of 1210 participants (575 mukbang viewers and 635 non-viewers) was included in the analysis (Figure A1).

### 2.3. Mukbang Viewing

To assess mukbang viewing status and frequency, participants were asked whether they had ever watched mukbang through social media platforms, such as YouTube and Instagram. Those who had prior viewing experience were then asked to indicate their viewing frequency over the preceding 12 months. Six initial response options were available: “Never,” “Less than once per week,” “1–2 times per week,” “3–4 times per week,” “5–6 times per week,” and “7 or more times per week.” For analytical purposes, the “5–6 times per week” and “7 or more times per week” categories were combined, resulting in five final groups: “Never,” “Less than once per week,” “1–2 times per week,” “3–4 times per week,” and “Five or more times per week.”

### 2.4. NQ (Nutrition Quotient)

This study utilized the NQ for adults to evaluate participants’ dietary behavior according to mukbang viewing frequency. The NQ for adults is a validated checklist used to assess overall dietary quality, nutritional status, and dietary behavior in adults aged 19–64 years [17]. It was developed in 2015 by the Korean Nutrition Society and Ministry of Food and Drug Safety and revised in 2021, culminating in the NQ-2021. The NQ-2021 comprises 18 questions categorized into three factors: Balance, Moderation, and Practice [18]. The Balance domain assesses the consumption frequency of various essential food groups, including vegetables, fruits, dairy products, fish, beans or bean products, nuts, mixed grains, and breakfast. The Moderation domain evaluates the restriction of unhealthy foods (e.g., greasy baked products or snacks, fast foods, spicy and salty soup foods, red meats, processed meats, etc.) and frequency of overeating and binge eating. The Practice domain examines adherence to healthy and safe eating behaviors. It includes the following items: “efforts to maintain healthy eating habits,” “checking nutrition labels,” “handwashing before meals,” and “the frequency of excessive alcohol consumption.” Each item is calculated using item specific scores and weights to derive domain scores, and the total NQ score is calculated from the domain scores using group specific weights [18,19]. All scores are scaled out of 100, with higher scores indicating superior dietary quality and behavior. Additionally, the NQ-2021 is classified into three categories “high”, “medium,” and “low” based on the established grade criteria. The total NQ score is categorized as follows: high (68.6–100), medium (52.8–68.5), and low (0–52.7). Each domain is also classified into three levels: Balance (high: 55.8–100, medium: 30.9–55.7, and low: 0–30.8), Moderation (high: 85.3–100, medium: 66.1–85.2, and low: 0–66.0), and Practice (high: 74.6–100, medium: 51.8–74.5, and low: 0–51.7) [18,19]. The medium and high categories were consolidated into a “medium–high” group for comparison with the low group.

### 2.5. Eating Behavior

To investigate changes in eating behavior following mukbang viewing, additional questions were exclusively posed to participants who had reported watching mukbang. Participants were initially asked, “Has there been a change in your dining out, delivery, or instant food consumption after watching mukbang?” Responses were categorized into three options: “No change,” “Increased,” and “Decreased.” They were also asked, “Have you ever experienced impulsive or binge eating after watching mukbang?”, with “Yes” and “No” as response options.

### 2.6. Covariates

Covariates included age (20–39; 40–64 years), sex (male; female), education level (high school or lower; college or higher), monthly household income (<3 million won, 3–6 million won, and ≥6 million won), household type (single-person household; multi-person household), drinking status (non-past drinkers; current drinkers), physical activity (moderate-to-vigorous intensity activity for 150 min/week, inactive, and active), weight control attempt (no; yes), health concern level (low, medium, and high), and body mass index (BMI) category (<18.5: underweight, 18.5–22.9: normal weight, 23–24.9: overweight, and ≥25 kg/m^2^: obese) [20]. These covariates were included to isolate independent associations between mukbang viewing and dietary quality. In particular, alcohol consumption, weight control attempts, and health concern level were included because they may be associated with both media consumption patterns and dietary behaviors, such as by influencing an individual’s motivation to watch food-related content as well as their food choices. Multicollinearity among the covariates was assessed using the variance inflation factor (VIF). All VIF values were less than 1.3, indicating no significant multicollinearity. These results are presented in Table A1.

### 2.7. Statistical Analysis

Descriptive statistics were conducted to compare sociodemographic variables, the NQ scores, and eating behavior between mukbang viewing groups using chi-squared tests for categorical variables and generalized linear regression for continuous variables. Multivariable logistic regression analyses were performed to analyze the relationships among mukbang viewing frequency, the NQ, and eating behavior. Odds ratios (ORs) with 95% confidence intervals (CIs) were estimated, adjusting for covariates, including age, sex, educational level, monthly household income, household type, alcohol drinking, physical activity, weight control attempt, health concern level, and obesity. *p* for trend was calculated by assigning the median value of each viewing category as a continuous variable in the regression model to assess linear trends across categories of mukbang viewing frequency. All analyses were conducted using SAS (version 9.4; SAS Institute Inc., Cary, NC, USA), and statistical significance was set at *p* < 0.05.

## 3. Results

### 3.1. Participant General Characteristics

Table 1 presents the participants’ characteristics according to mukbang viewing frequency. Frequent mukbang viewers were more likely to be younger, have higher levels of education and monthly household income, be current drinkers, be more physically active, be more likely to attempt weight control, and have greater health concerns compared with mukbang non-viewers (*p* < 0.05). In contrast, no significant differences were observed in sex, household type, or BMI category with mukbang viewing frequency.

### 3.2. NQ According to Mukbang Viewing Frequency

NQ-2021 scores and grades by mukbang viewing frequency are presented in Table 2 and Table A2. Compared with non-viewers, frequent mukbang viewers had higher scores in the Balance domain and were more frequently classified into the medium–high grade. Item-level analysis within the Balance domain demonstrated that frequent viewers had significantly higher scores for all items, except mixed grain and breakfast consumption (*p* < 0.05). In contrast, within the Moderation domain, a higher mukbang viewing frequency was associated with lower scores, and a greater proportion of viewers were classified into the low grade. Further analysis revealed that frequent viewers had significantly lower scores across all Moderation items than non-viewers (*p* < 0.001). While no significant differences were observed in the total NQ score or the Practice domain. A detailed item-level analysis of the Practice domain indicated that frequent mukbang viewers scored higher on items, such as “efforts to maintain healthy eating habits” and “checking nutrition labels”. Conversely, they exhibited scored lower scores on alcohol related items than non-viewers.

### 3.3. ORs for NQ Grade According to Mukbang Viewing Frequency

Table 3 presents the association between mukbang viewing frequency and NQ grade based on ORs adjusted for sociodemographic variables. In the Balance domain, a significant positive trend was observed, with a higher viewing frequency associated with increased odds of being in the medium–high NQ grade (*p* for trend < 0.001). In particular, those who viewed mukbang most frequently (≥5 times/week) had the highest odds of being in the medium–high NQ grade compared with non-viewers. Conversely, the Moderation domain showed an inverse relationship, with higher mukbang viewing frequency associated with significantly lower odds of being in the medium–high NQ grade (*p* for trend < 0.001). The lowest odds were observed in the “3–4 times/week” group, followed by the “≥5 times/week” group, relative to the non-viewer group. No significant associations were observed in the Practice domain or total NQ score.

### 3.4. ORs for Eating Behaviors According to Mukbang Viewing Frequency

To examine the association between mukbang viewing frequency and eating behavior changes, both descriptive statistics and logistic regression analyses were employed. The distribution of eating behavior changes by viewing frequency is presented in Table A3, and the OR results for this association are shown in Table 4.

Table A3 presents the distribution of changes in dining out, delivery, or instant food consumption, and impulsive or binge eating behavior, according to mukbang viewing frequency. Significant proportional differences were observed among mukbang viewing frequency groups for both variables (*p* < 0.001 for both). Regarding changes in frequency of dining out, delivery, or instant food consumption, a greater proportion of participants in the “≥5 times/week” group reported an increase in dining out, delivery, or instant food consumption frequency (66.2%) than that in the “<1 time/week” group (37.6%). With regard to impulsive eating behavior, within each group, a larger proportion of frequent mukbang viewers (55.4% in the “≥5 times/week” group) reported impulsive eating than in the “<1 time/week” group (29.2%).

The ORs of the association between mukbang viewing frequency and eating behavior are presented in Table 4. After adjusting for sociodemographic variables, mukbang viewing frequency was significantly associated with dining out, delivery, or instant food consumption, and impulsive or binge eating behavior. Compared with individuals who viewed mukbang less than once a week (<1 time/week), those in the “≥5 times/week” group exhibited the highest odds of reporting an increase (OR = 3.24, 95% CI: 1.72–6.08). Overall, mukbang viewing frequency was not significantly associated with a decrease in dining out, delivery, or instant food consumption behaviors. However, individuals in the “3–4 times/week” group had significantly higher odds of reporting a decrease than those in the “<1 time/week” group (OR = 3.55, 95% CI: 1.42–8.90). Similarly, a higher mukbang viewing frequency was associated with increased odds of impulsive or binge eating behavior, showing a dose–response pattern across viewing frequency groups.

## 4. Discussion

This study examined the association of mukbang viewing frequency with NQ scores and eating behavior change among Korean adults. A higher mukbang viewing frequency was associated with higher Balance domain scores and lower Moderation domain scores than non-viewing. However, Practice domain and total NQ scores were not associated with mukbang viewing frequency. Additionally, significant associations were observed between mukbang viewing frequency and dining out, delivery, or instant food consumption, and impulsive or binge eating. Compared with those in the “<1 time/week” group, individuals who viewed mukbang more frequently exhibited a greater association with these dietary behaviors.

In this study, no significant differences were observed in the total NQ score according to mukbang viewing frequency. However, this may be attributed to opposing trends across individual domains offsetting each other in the overall average. Notably, domain-specific analyses revealed contradictory patterns depending on viewing frequency.

In the Balance domain, the scores were significantly higher in the high-viewing-frequency groups, and a significant increase in the detailed items of vegetable and fruit intake was also confirmed. This finding may be partially explained by the nature of mukbang content. Mukbang often features a wide range of foods—from everyday meals to unfamiliar or culturally diverse dishes—which may stimulate viewers’ curiosity and appetite [21]. This exposureis potentially linked to greater dietary variety and interest in trying new foods, particularly among frequent viewers. This result contrasts with previous findings wherein adults with longer mukbang viewing times reported lower preferences for vegetables and fruits [11], and adolescent viewers of mukbang and cookbang content tended to consume fewer fruits and vegetables [4].

In contrast, Moderation domain scores were significantly lower among more frequent mukbang viewers. This is attributable to mukbang content’s predominant focus on high-calorie, low-nutrient foods (e.g., instant foods, late-night snacks, and fast foods) and frequent inclusion of overeating as a major factor [1,12]. Previous studies have also reported associations between mukbang viewing and increased consumption of unhealthy foods, including fast foods, snacks, sweets, and late-night meals, and the current findings are consistent with this trend [4,12,13,14]. However, the NQ-2021 does not include a specific item for sweet foods, possibly leading to an underestimation of the related dietary impacts.

These divergent results highlight a ‘Mukbang Paradox’ observed in this study, in which frequent mukbang viewing was associated with both improved dietary balance and impaired moderation. This pattern may reflect a dual mechanism whereby increased interest in food coexists with reduced self-regulation in response to strong visual and sensory stimulation. Specifically, while greater interest in food may encourage dietary variety and information-seeking behaviors, the highly stimulating nature of mukbang content may also be associated with excessive intake. Taken together, this paradox provides an important context for interpreting the patterns observed in the Practice domain.

While no significant differences were observed in the total scores of the Practice domain according to mukbang viewing frequency, item-level analysis revealed that more frequent viewers scored higher in “efforts to maintain healthy eating habits” and “checking nutrition labels” but scored lower in drinking. Indeed, previous studies have demonstrated that individuals with a high interest in food are more likely to engage with mukbang content [22], and those with greater food involvement demonstrate more health-conscious food choices [23]. Additionally, adolescents with a high interest in food are more likely to check the label and consider nutritional value when purchasing food [10]. Individuals with strong food interest also tend to seek and retain more food-related information, further promoting informed and intentional dietary practices. These findings support the possibility that mukbang viewers’ greater interest in food might have contributed to the higher scores observed in specific Practice items. This pattern potentially reflects a dual mechanism: while greater food interest encourages informed dietary practices, frequent exposure to unhealthy eating through mukbang is observed alongside compensatory health behaviors.

Consistent with this, the theory of Compensatory Health Beliefs (CHBs) posits that individuals may believe that the negative effects of unhealthy behaviors can be offset by subsequent healthy behavior [24]. This suggests that the activation of CHBs may contribute to the inconsistency between health intentions and actual dietary behaviors [25,26]. However, the underlying mechanisms remain unclear, and further research is required to more comprehensively elucidate the impact of mukbang viewing on dietary quality and eating behaviors.

Meanwhile, interesting results were also derived from changes in eating behavior. The higher the frequency of mukbang viewing, the greater the frequency of dining out, delivery, or instant food consumption, and impulsive or binge eating. This is consistent with the results of previous studies targeting adolescents and adults [11,14,15,27]. According to previous studies, mukbang content frequently includes unhealthy eating behaviors, such as binge eating, excessive eating, rapid consumption, and the intake of highly stimulating foods [1,28,29]. In addition, while some content may feature various dishes, the most commonly showcased foods tend to be high in calories, poor in nutrients, and high in salt and sugar (e.g., processed foods, delivery foods, and fast foods) [28,29]. Exposure to mukbang content may increase viewers’ appetite as well as encourage uncontrolled eating or mimicry of high-calorie food consumption and unhealthy behaviors frequently depicted in such videos [30]. Repeated exposure to mukbang content may lead viewers to perceive unhealthy eating patterns as normal behaviors [13]. Moreover, seeing mukbang hosts maintain a lean appearance despite consuming vast quantities of food may promote distorted beliefs regarding weight control [6,7]. These findings suggest that mukbang viewing may be associated with adverse eating behaviors and potentially linked to negative health outcomes, including obesity and disordered eating [6]. Furthermore, they highlight the importance of public health interventions, including the appropriate regulation of mukbang content, as well as educational efforts that help viewers interpret and respond to such content in a healthy and informed manner.

Despite these risks, not all frequent viewers followed this pattern. Interestingly, the group watching mukbang 3–4 times a week showed the highest odds of decreased dining out, delivery, or instant food consumption, suggesting alternative behavioral motivations. This indicates that certain individuals may vicariously use mukbang as a substitute for actual eating, potentially owing to dieting and weight management [7,31]. Moreover, beyond its potential risks, mukbang may offer vicarious satisfaction, satiety, and pleasure; alleviate feelings of social isolation and loneliness; and provide information regarding food, nutrition, and cooking [4,6,7,32]. Considering these contrasting patterns, public health approaches should be twofold—regulating harmful content while encouraging formats that promote dietary awareness and healthy practices. In particular, mukbang platforms with widespread influence may help encourage positive dietary behaviors.

This study has certain limitations. First, because of its cross-sectional design, causal relationships between mukbang viewing and dietary outcomes could not be established. The observed associations may reflect reverse causation, as individuals with particular dietary behaviors may be more likely to engage in mukbang viewing, rather than mukbang viewing leading to changes in dietary behaviors. Second, all data were self-reported, including viewing frequency, NQ scores, and dietary behaviors, possibly subjecting participants to social desirability bias. Third, this study did not account for mukbang content type and participants’ motivations for viewing mukbang, aspects that could have influenced the results. According to previous studies, viewing motivation may affect eating habits and the NQ score in Korean adolescents [33]. Despite these limitations, this study has notable strengths. It is one of the few studies to investigate the relationship between mukbang viewing and dietary behaviors in a general adult population, rather than focusing solely on adolescents. By comparing mukbang viewers with non-viewers, this study could more clearly assess the potential impact of mukbang content. Furthermore, NQ scores and eating behaviors according to viewing frequency facilitated the investigation of how the extent of mukbang consumption may be associated with actual dietary habits.

## 5. Conclusions

In conclusion, this study revealed that mukbang viewing frequency is significantly associated with NQ scores and dietary behaviors in Korean adults. A higher mukbang viewing frequency was related to greater dietary diversity, as reflected by the higher scores in the Balance domain and lower scores in the Moderation domain, indicating the increased consumption of unhealthy foods. Furthermore, more frequent viewing was associated with a greater frequency of dining out, delivery, or instant food consumption, and impulsive or binge eating. These findings suggest that mukbang viewing is associated with both beneficial and adverse dietary behaviors among adults. Considering mukbang’s widespread popularity, strategies and public health interventions that encourage healthy mukbang content are crucial.

## Figures and Tables

**Table 1:** Study population’s general characteristics according to mukbang viewing frequency.

	Frequency of Watching Mukbang ^(1)^					
	Never	<1 Time/Week	1–2 Times/Week	3–4 Times/Week	≥5 Times/Week	*p*-Value ^(2)^
**Total**	635 (52.5)	202 (16.7)	211 (17.4)	88 (7.3)	74 (6.1)	
**Age**						
20–39 years	239 (37.6)	60 (29.7)	89 (42.2)	43 (48.9)	43 (58.1)	<0.001
40–64 years	396 (62.4)	142 (70.3)	122 (57.8)	45 (51.1)	31 (41.9)	
**Sex**						
Male	326 (51.3)	97 (48.0)	119 (56.4)	49 (55.7)	39 (52.7)	0.475
Female	309 (48.7)	105 (52.0)	92 (43.6)	39 (44.3)	35 (47.3)	
**Education level**						
High school or lower	139 (21.9)	36 (17.8)	28 (13.3)	11 (12.5)	10 (13.5)	0.017
College or higher	496 (78.1)	166 (82.2)	183 (86.7)	77 (87.5)	64 (86.5)	
**Monthly household income**						
<3 million won	193 (30.4)	49 (24.3)	38 (18.0)	16 (18.2)	11 (14.9)	0.001
3–6 million won	278 (43.8)	90 (44.6)	101 (47.9)	44 (50.0)	32 (43.2)	
≥6 million won	164 (25.8)	63 (31.2)	72 (34.1)	28 (31.8)	31 (41.9)	
**Household type**						
Single-person household	113 (17.8)	35 (17.3)	36 (17.1)	17 (19.3)	11 (14.9)	0.960
Multi-person household	522 (82.2)	167 (82.7)	175 (82.9)	71 (80.7)	63 (85.1)	
**Drinking status**						
Non-past drinker	236 (37.2)	45 (22.3)	56 (26.5)	20 (22.7)	12 (16.2)	<0.001
Current drinker	399 (62.8)	157 (77.7)	155 (73.5)	68 (77.3)	62 (83.8)	
**Physical activity ^(^** **^3)^**						
Inactive	585 (92.1)	186 (92.1)	191 (90.5)	81 (92.0)	60 (81.1)	0.033
Active	50 (7.9)	16 (7.9)	20 (9.5)	7 (8.0)	14 (18.9)	
**Weight control attempt**						
No	329 (51.8)	96 (47.5)	84 (39.8)	38 (43.2)	22 (29.7)	0.001
Yes	306 (48.2)	106 (52.5)	127 (60.2)	50 (56.8)	52 (70.3)	
**Health concern**						
Low	80 (12.6)	19 (9.4)	23 (10.9)	9 (10.2)	3 (4.1)	<0.001
Medium	286 (45.0)	81 (40.1)	61 (28.9)	39 (44.3)	28 (37.8)	
High	269 (42.4)	102 (50.5)	127 (60.2)	40 (45.5)	43 (58.1)	
**BMI category ^(4)^**						
Underweight	37 (5.8)	14 (6.9)	16 (7.6)	7 (8.0)	7 (9.5)	0.514
Normal weight	272 (42.8)	86 (42.6)	94 (44.6)	38 (43.2)	36 (48.7)	
Overweight	140 (22.1)	55 (27.2)	53 (25.1)	18 (20.5)	18 (24.3)	
Obese	186 (29.3)	47 (23.3)	48 (22.7)	25 (28.4)	13 (17.6)	

^(1)^ Values are presented as N (%). ^(2)^ *p*-values were obtained using chi-squared tests for categorical variables. ^(3)^ Physical activity was defined as moderate-to-vigorous intensity activity for 150 min/week. ^(4)^ BMI: body mass index. BMI category (<18.5: underweight, 18.5–22.9: normal weight, 23–24.9: overweight, and ≥25 kg/m^2^: obese).

**Table 2:** NQ scores and frequency of NQ grade according to mukbang viewing frequency.

	Frequency of Watching Mukbang ^(1)^					
	Never	<1 Time/Week	1–2 Times/Week	3–4 Times/Week	≥5 Times/Week	*p*-Value ^(2)^
**Balance**	34.4 ± 19.2	38.4 ± 19.0	37.7 ± 16.2	40.7 ± 17.1	48.1 ± 22.6	<0.001
Medium to High **^(3)^**	338 (53.2)	121 (59.9)	137 (64.9)	63 (71.6)	57 (77.0)	<0.001
Low	297 (46.8)	81 (40.1)	74 (35.1)	25 (28.4)	17 (23.0)	
**Moderation**	71.4 ± 15.5	68.7 ± 13.9	65.0 ± 14.2	56.3 ± 15.0	52.4 ± 21.6	<0.001
Medium to High	406 (63.9)	122 (60.4)	94 (44.5)	21 (23.9)	19 (25.7)	<0.001
Low	229 (36.1)	80 (39.6)	117 (55.5)	67 (76.1)	55 (74.3)	
**Practice**	60.1 ± 16.0	61.7 ± 14.7	63.3 ± 17.3	60.5 ± 16.6	62.7 ± 15.0	0.111
Medium to High	442 (69.6)	153 (75.7)	159 (75.4)	62 (70.5)	56 (75.7)	0.288
Low	193 (30.4)	49 (24.3)	52 (24.6)	26 (29.5)	18 (24.3)	
**Total NQ ^(4)^**	55.8 ± 11.6	56.8 ± 11.3	56.1 ± 11.6	53.3 ± 10.5	55.2 ± 11.2	0.193
Medium to High	357 (56.2)	125 (61.9)	124 (58.8)	43 (48.9)	41 (55.4)	0.304
Low	278 (43.8)	77 (38.1)	87 (41.2)	45 (51.1)	33 (44.6)	

^(1)^ Values are presented as the mean ± SD or N (%). ^(2)^ *p*-values were obtained using chi-squared tests for categorical variables and generalized linear regression for continuous variables. ^(3)^ Grade criteria were obtained from total scores from the revised Nutrition Quotient for Korean adults (NQ-2021): medium to high: 25% percentile ≤ NQ-2021; low: NQ-2021 < 25% percentile. ^(4)^ NQ, Nutrition Quotient.

**Table 3:** Odds ratios and 95% confidence intervals of NQ grade according to mukbang viewing frequency.

	Frequency of Watching Mukbang					
	Never	<1 Time/Week	1–2 Times/Week	3–4 Times/Week	≥5 Times/Week	*p* for Trend ^(2)^
**Balance**	1.00 ^(1),(3),(4)^	1.16 (0.82–1.64)	1.50 (1.05–2.12)	2.56 (1.53–4.31)	2.88 (1.57–5.30)	<0.001
**Moderation**	1.00	0.83 (0.59–1.17)	0.45 (0.33–0.65)	0.19 (0.11–0.33)	0.21 (0.12–0.38)	<0.001
**Practice**	1.00	1.38 (0.91–2.07)	1.22 (0.81–1.83)	1.09 (0.63–1.90)	1.20 (0.64–2.25)	0.211
**Total NQ ^(^** **^5)^**	1.00	1.20 (0.83–1.73)	0.99 (0.68–1.42)	0.79 (0.48–1.30)	0.83 (0.47–1.46)	0.119

^(1)^ Values are expressed as odds ratio (95% confidence interval). ^(2)^ *p* for trend was calculated by assigning the median value of each mukbang viewing frequency category as a continuous variable in the regression model. ^(3)^ Odds ratios represent the likelihood of being in the “Medium to High” NQ grade group, with the “Low” grade group serving as the reference. Grade criteria were obtained from total scores from the revised Nutrition Quotient for Korean adults (NQ-2021): medium to high: 25% percentile ≤ NQ-2021; low: NQ-2021 < 25% percentile. ^(4)^ Odds ratios were adjusted for age, sex, educational level, monthly household income, household type, alcohol drinking, physical activity, weight control attempt, health concern level, and obesity. ^(5)^ NQ, Nutrition Quotient.

**Table 4:** Odds ratios and 95% confidence intervals of eating behaviors according to mukbang viewing frequency.

	Frequency of Watching Mukbang			
	<1 Time/Week	1–2 Times/Week	3–4 Times/Week	≥5 Times/Week
**Changes in the frequency of dining out, delivery, or instant food consumption**				
No change	1.00 ^(1),(2)^	1.00	1.00	1.00
Increase	1.00	1.59 (1.04–2.43)	2.71 (1.53–4.82)	3.24 (1.72–6.08)
Decrease	1.00	1.53 (0.69–3.41)	3.55 (1.42–8.90)	2.30 (0.72–7.30)
**Impulsive or binge eating behavior**				
No	1.00	1.00	1.00	1.00
Yes	1.00	1.93 (1.25–2.99)	2.72 (1.57–4.73)	2.80 (1.55–5.06)

^(1)^ Values are expressed as odds ratio (95% confidence interval). ^(2)^ Odds ratios were adjusted for age, sex, educational level, monthly household income, household type, alcohol drinking, physical activity, weight control attempt, health concern level, and obesity.

## Data Availability

The data presented in this study are available on request from the corresponding authors due to privacy restrictions related to internal survey data collected by the National Cancer Center.

## Abbreviations

The following abbreviations are used in this manuscript:

BMI	Body Mass Index
CHBs	Compensatory Health Beliefs
Cis	Confidence intervals
NQ	Nutrition Quotient
ORs	Odds Ratios

## Appendix A

**Table A1:** Multicollinearity assessment of covariates (VIF).

Variable	VIF
Mukbang viewing frequency	1.07
Age	1.15
Sex	1.19
Education level	1.11
Monthly household income	1.29
Household type	1.28
Drinking status	1.06
Physical activity	1.05
Weight control attempt	1.12
Health concern	1.09
BMI category	1.22

**Table A2:** NQ item scores according to mukbang viewing frequency.

	Frequency of Watching Mukbang					
	Never	<1 Time/Week	1–2 Times/Week	3–4 Times/Week	≥5 Times/Week	*p*-Value
**Total**	55.8	56.8	56.1	53.3	55.2	0.193
**Balance**	34.4	38.4	37.7	40.7	48.1	<0.001
Number of vegetable dishes, excluding Kimchi, at each meal	40.6	45.7	42.5	45.2	52.0	0.002
Intake frequency of fruits	34.5	38.6	35.7	38.6	49.0	0.001
Intake frequency of milk or dairy products	29.8	35.0	35.2	37.2	45.6	<0.001
Intake frequency of fishes	23.3	29.2	30.2	34.1	43.6	<0.001
Intake frequency of beans or bean products	24.5	28.7	29.9	32.1	39.5	<0.001
Intake frequency of nuts	29.5	35.0	34.7	40.3	49.0	<0.001
Intake frequency of cooked rice with mixed grains	38.5	42.5	39.6	40.1	49.7	0.064
Intake frequency of breakfast	43.5	43.6	46.5	50.3	50.7	0.388
**Moderation**	71.4	68.7	65.0	56.3	52.4	<0.001
Intake frequency of greasy baked products or snacks	69.8	66.3	60.3	51.1	49.7	<0.001
Intake frequency of fast foods	77.0	75.0	66.9	58.0	55.1	<0.001
Intake frequency of spicy and salty soup and stew	73.5	71.4	69.0	58.8	57.8	<0.001
Intake frequency of red meats	60.7	54.3	56.4	52.8	44.6	<0.001
Intake frequency of processed meats	73.5	73.0	68.6	58.8	56.1	<0.001
Frequency of overeating or binge eating	71.9	69.3	67.0	57.0	51.4	<0.001
**Practice**	60.1	61.7	63.3	60.5	62.7	0.111
Efforts to have healthy eating habits	55.3	60.5	62.2	57.7	63.5	<0.001
Nutrition labeling check when eating out or purchasing processed foods	42.7	47.3	53.6	52.8	59.1	<0.001
Washing hands practices before eating meals	72.6	73.5	73.7	73.0	78.4	0.342
Heavy drinking frequency of alcohol	75.8	68.0	65.9	63.0	51.1	<0.001

**Table A3:** Frequency of eating habits according to mukbang viewing frequency.

	Frequency of Watching Mukbang				
	<1 Time/Week	1–2 Times/Week	3–4 Times/Week	≥5 Times/Week	*p*-Value
**Changes in the frequency of dining out, delivery, or instant food consumption**					
No change	113 (56.0)	93 (44.1)	26 (29.5)	19 (25.7)	<0.001
Increase	76 (37.6)	100 (47.4)	49 (55.7)	49 (66.2)	
Decrease	13 (6.4)	18 (8.5)	13 (14.8)	6 (8.1)	
**Impulsive or binge eating behavior**					
No	143 (70.8)	123 (58.3)	43 (48.9)	33 (44.6)	<0.001
Yes	59 (29.2)	88 (41.7)	45 (51.1)	41 (55.4)	
